# Determining the Functions of HIV-1 Tat and a Second Magnesium Ion in the CDK9/Cyclin T1 Complex: A Molecular Dynamics Simulation Study

**DOI:** 10.1371/journal.pone.0124673

**Published:** 2015-04-24

**Authors:** Hai-Xiao Jin, Mei-Lin Go, Peng Yin, Xiao-Ting Qiu, Peng Zhu, Xiao-Jun Yan

**Affiliations:** 1 Key Laboratory of Applied Marine Biotechnology Ministry of Education, School of Marine Sciences, Ningbo University, Ningbo, China; 2 Department of Pharmacy, National University of Singapore, Singapore, Singapore; 3 Key Laboratory of Chemical Biology and Traditional Chinese Medicine Research (Ministry of Education), College of Chemistry and Chemical Engineering, Hunan Normal University, Changsha, China; George Mason University, UNITED STATES

## Abstract

The current paradigm of cyclin-dependent kinase (CDK) regulation based on the well-established CDK2 has been recently expanded. The determination of CDK9 crystal structures suggests the requirement of an additional regulatory protein, such as human immunodeficiency virus type 1 (HIV-1) Tat, to exert its physiological functions. In most kinases, the exact number and roles of the cofactor metal ions remain unappreciated, and the repertoire has thus gained increasing attention recently. Here, molecular dynamics (MD) simulations were implemented on CDK9 to explore the functional roles of HIV-1 Tat and the second Mg^2+^ ion at site 1 (Mg_1_^2+^). The simulations unveiled that binding of HIV-1 Tat to CDK9 not only stabilized hydrogen bonds (H-bonds) between ATP and hinge residues Asp104 and Cys106, as well as between ATP and invariant Lys48, but also facilitated the salt bridge network pertaining to the phosphorylated Thr186 at the activation loop. By contrast, these H-bonds cannot be formed in CDK9 owing to the absence of HIV-1 Tat. MD simulations further revealed that the Mg_1_^2+^ ion, coupled with the Mg_2_^2+^ ion, anchored to the triphosphate moiety of ATP in its catalytic competent conformation. This observation indicates the requirement of the Mg_1_^2+^ ion for CDK9 to realize its function. Overall, the introduction of HIV-1 Tat and Mg_1_^2+^ ion resulted in the active site architectural characteristics of phosphorylated CDK9. These data highlighted the functional roles of HIV-1 Tat and Mg_1_^2+^ ion in the regulation of CDK9 activity, which contributes an important complementary understanding of CDK molecular underpinnings.

## Introduction

Cyclin-dependent kinase 9 (CDK9) is a Ser/Thr kinase that belongs to the family of cyclin-dependent kinases (CDKs). CDK9 serves as the catalytic subunit of the positive transcription elongation factor b (P-TEFb; CDK9/cyclin T), which phosphorylates the RNA polymerase II C-terminal domain and the negative elongation factors NELF and DRB (dichlorobenzimidazole riboside)-sensitivity-inducing factor (DSIF) to trigger the elongation of many gene transcripts [1]. P-TEFb has been an important therapeutic target in oncology, virology, and cardiology [2,3]. A viral protein, human immunodeficiency virus type 1 (HIV-1) Tat, interacts with P-TEFb and induces the factor to promote the productive elongation of HIV mRNA [4–6]. Biochemical experiments have shown that Tat increased transcriptional elongation performed by CDK9 [7].

**Fig 1:**
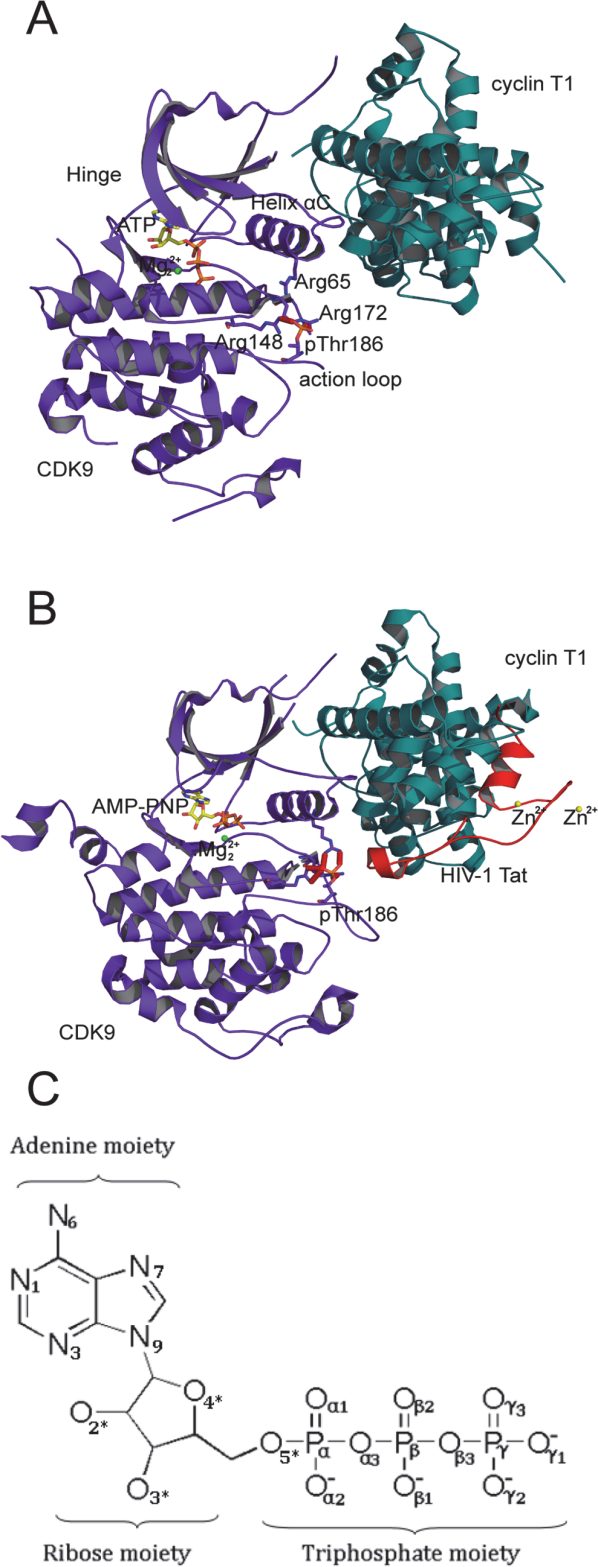
Architecture of CDK9/cyclin T1. (A) Ribbon representation of the overall crystal structure of pCDK9/cyclin T1 bound to ATP and one Mg^2+^ ion at site 2 (Mg_2_^2+^ ion) complex (PDB code 3BLQ). (B) Ribbon representation of the overall crystal structure of pCDK9/cyclin T1 bound to HIV-1 Tat and AMP-PNP and one Mg_2_^2+^ ion (PDB code 3MIA). CDK9 is purple blue, cyclin T1 is deep teal, and HIV-1 Tat is red. ATP, AMP-PNP, and pThr186 are drawn as sticks, and Zn^2+^ ions and Mg^2+^ ions are drawn as yellow and green spheres, respectively. The salt bridge network formed by pThr186 is shown as a red dotted line. (C) Structure of ATP with labeled oxygen and nitrogen atoms used in this article.

Twenty CDK9 crystal structures have thus far been solved [8–18], and their availability serves as a valuable resource for structure-aided drug design. CDK9 adopts a typical bilobal fold (Fig 1A), which is extremely conserved among Ser/Thr and Tyr kinases. The N-terminal lobe is composed of a five-stranded antiparallel β-sheet and one prominent α-helix, i.e., the helix αC (sequence PITALRE in CDK9 and PSTAIRE in CDK2). The larger C-terminal lobe is mostly helical and connected to the N-terminal lobe by the so-called flexible hinge region (residues 104–107). ATP is sandwiched between the N- and C-terminal lobes and anchors its adenine moiety by H bonds with Asp104 and Cys106 in the hinge region. Cyclin T has a canonical cyclin structure. The interface of CDK9/cyclin T is notably smaller than that of the CDK2/cyclin A complex and is restricted to the N-terminal lobe of CDK9.

Although a few kinases are known to harness a single divalent ion or none at all[19], many, if not all, protein kinases require two divalent metal ions for optimum catalysis [20]. In the kinome, the exact number and roles of the cofactor metal ions remain unknown, and the repertoires have recently gained increasing attention. Thus far, a few CDK9 crystal structures are associated with a single Mg^2+^ ion at site 2 (Mg_2_^2+^ ion; Fig 1A and 1B), whereas the other Mg^2+^ ion at site 1 (Mg_1_^2+^ ion) is absent in all CDK9 crystal structures. The crystal structures of CDK2 with two Mg^2+^ ions had not been solved until recently, when the crystal structures of CDK2/cyclin A transition state (TS) complex with two Mg^2+^ ions were discovered by Yong et al. [21,22]. Experimental observations have demonstrated that Mg^2+^ concentration could represent an important regulator of CDK2 activity in vivo [21]. These findings raise an interesting question on whether CDK9 requires the binding of two Mg^2+^ ions to realize its function, as in the case of CDK2.

CDK2 is a phenotypic family of CDKs. A series of CDK2/cyclin A structures have provided significant insight into the molecular underpinnings of CDK activation and regulation. The need of CDKs to be associated with a cognate cyclin, followed by phosphorylation on threonine at the activation loop to realize full activity, is well-documented [23,24]. However, the generality of this underlying mechanism has been challenged with the determination of new CDK crystal structures. For instance, the crystal structures of CDK4/cyclin D revealed that cyclin D binding and activation loop phosphorylation do not adequately enable the CDK4 to adopt an active conformation [24,25]. In fact, recent experiments underscored that association with additional protein substrates and/or cofactor binding are critical to the remodeling of CDK4/cyclin D into its active conformation [25–27]. Additionally, the solved CDK9/cyclin T crystal structures demonstrated some notable structural differences in the phosphorylated Thr186 (pThr186) at the activation loop. Although the phosphorylation of Thr186 is required for CDK9 activation [28], pThr186 in CDK9/cyclin T1 complex crystal structures only forms salt bridge interactions with Arg148 and Arg172. This condition is unlike that the CDK2/cyclin A complex, in which pThr160 forms salt bridge interactions with the positively charged triad of arginine residues (Arg50, Arg126, and Arg150). pThr186 is slightly distant (3.9 Å) from the third arginine residue (Arg65), which is located in the αC helix (Fig 1A) [8]. Interestingly, as shown in Fig 1B, the crystal structure of CDK9/cyclin T1 in a complex with a minimal activation domain (residues 1–48) of HIV-1 Tat formed five salt bridges with the arginine triad [9]. HIV-1 Tat, a two-zinc-mediated viral transactivator of transcription, inserts itself into a groove at the heterodimer interface, thus augmenting interactions and resulting in a more stable P-TEFb complex. The reestablished Arg65–pThr186 ion pair results in a more stable local conformation and contributes to maintaining strong contact between the critical activation loop and the prominent αC helix motif. The current understanding of kinase regulation owes much to X-ray crystallography [29]. In this study, we performed 50 ns explicit-solvent MD simulations on five different systems to determine the influence of HIV-1 Tat binding, activation loop phosphorylation, and the presence of Mg_1_^2+^ at site 1 on CDK9 dynamics. Our results revealed elaborate and significant differences in the dynamics behavior of CDK9, which provides insight into the current understanding of CDK regulation and may contribute to structure-based drug design.

## Materials and Methods

### Initial Complexes Preparation

The crystal structure of phosphorylated CDK9/cyclin T1 complex bound to ATP and one Mg^2+^ ion at site 2 (Mg_2_^2+^ ion) (pCDK9/cyclin T1/ATP/1MG complex) was obtained from the RCSB Protein Data Bank (PDB code 3BLQ) [8] (Fig 1A). The first four simulation systems were based on this crystal structure. The missing Mg^2+^ ion at site 1 (Mg_1_^2+^ ion) was modeled into its position by aligning the crystal structure of GSK3β in the complex with ATP analog adenosine 5′-(β,γ-imidotriphosphate) (AMP-PNP) and two Mg^2+^ ions (PDB code 1PYX) [30] with the pCDK9/cyclin T1/ATP/1MG complex crystal structure. The Mg_1_^2+^ ion is and subsequently extracted to model the pCDK9/cyclin T1/ATP/2MG complex. The pThr186 at the active loop of the two complexes was replaced with nonphosphorylated Thr186 to construct the CDK9/cyclin T1/ATP/1MG and CDK9/cyclin T1/ATP/2MG complexes. The fifth simulated complex was based on another crystal structure of pCDK9/cyclin T1 in the complex with AMP-PNP and Mg_2_^2+^ ion and the minimal activation domain (residues 1–48) of HIV-1 Tat (pCDK9/cyclin T1/AMP-PNP/1MG/Tat complex; PDB code 3MIA) [9] (Fig 1B). The missing Mg_1_^2+^ ion in the X-ray structure results in the lack of a coordination bond between the ATP O_γ_ atom and Mg_1_^2+^ ion, which causes the γ-phosphate of AMP-PNP to assume an unusual conformation. Therefore, the crystal structure of the GSK3β/AMP-PNP/2MG complex was also aligned with the crystal structure of the pCDK9/cyclin T1/AMP-PNP/1MG/Tat complex to extract both AMP-PNP and Mg_1_^2+^ ion coordinates into the pCDK9 ATP binding pocket. The imido nitrogen atom was subsequently replaced with the oxygen atom, thus generating the pCDK2/cyclin T1/ATP/2MG/Tat complex. The missing residues in the two CDK9 X-ray structures were completed using Molecular Operating Environment [31] and further minimized by AMBER 11 [32] in the subsequent procedure. The detailed compositions of the five initial complexes are summarized in Table 1.

**Table 1:** The compositions of the five simulations.

Complex	System code	Phosphorylaton state	Composition
CDK9/cyclin T1/ATP/1Mg	1	Thr186	Dephosphorylated CDK9, cyclin T1, ATP, Mg_2_^2+^
pCDK9/cyclin T1/ATP/1Mg	2	pThr186	Phosphorylated CDK9, cyclin T1, ATP, Mg_2_^2+^
CDK9/cyclin T1/ATP/2Mg	3	Thr186	Dephosphorylated CDK9, cyclin T1, ATP, Mg_1_^2+^, Mg_2_^2+^
pCDK9/cyclin T1/ATP/2Mg	4	pThr186	Phosphorylated CDK9, cyclin T1, ATP, Mg_1_^2+^, Mg_2_^2+^
pCDK9/cyclin T1/ATP/2Mg/Tat	5	pThr186	Phosphorylated CDK9, cyclin T1, ATP, Mg_1_^2+^, Mg_2_^2+^, HIV-1 Tat

### Force Field

The AMBER force field (ff99SB) [33] was applied to the CDK9 protein, cyclin T1 protein, HIV-1 Tat protein, and Mg^2+^ ions. Glu/Asp residues were deprotonated and Lys/Arg residues were protonated at a simulated pH of 7. The protonated states for the His residues were assigned according to the PROPKA calculation, with the exception of the involvement of those in the coordination with the zinc ion [His33 in the HIV-1 Tat coordinated to the zinc ion was modeled as a negative charge state (−1 charge)]. We adopted the cationic dummy atom approach introduced by Pang et al. [34,35] to describe the zinc divalent cation. This approach places four cationic dummy atoms in a tetrahedral arrangement in the zinc nucleus to mimic the 4s4p^3^ vacant orbitals of the zinc ion. Thus, the lone-pair electrons of the zinc coordinates occupy the vacant orbitals and fulfill the orientation requirements for the tetrahedral coordination geometry of zinc. The zinc nucleus was assigned with only van der Waals parameters (van der Waals r* = 3.1 Å, van der Waals potential well depth ε = 1E-6 kcal/mol, and charge *q* = 0), whereas the dummy atom was assigned only with charge (r* = 0, *ε* = 0, and *q* = 0.5e). The force field parameters for the -2 charged pThr186 and the -4 charged ATP were taken from the AMBER parameter database [36,37].

### MD Simulations

The hydrogen atoms and the missing atoms were added to the Leap module of AMBER 11 [32]. Each system was immersed in the truncated octahedron box of TIP3P [38] water molecules with a 10 Å buffer in each direction. An appropriate number of Cl^-^ counterions were then added through the random substitution of solvent water molecules with Cl^-^ ions at the most favorable electrostatic potential positions to maintain the electroneutrality of the five systems. Thus, the total number of atoms for Systems 1, 2, 3, 4, and 5 were 68110, 68297, 68308, 68309, and 93339, respectively. Energy minimizations and MD simulations were conducted using the SANDER module of AMBER 11 with periodic boundary conditions. Prior to the production run, each system was optimized by using three steps minimization. First, ATP, metal ions, and protein residues were fixed with harmonic force restraint, and only the positions of the water molecules were minimized. Second, ATP, metal ions, and protein residues from crystal structures were constrained, whereas the added missing residues and water molecules were minimized. Finally, the whole system was allowed to fully relax. In each step, energy minimization was performed using the steepest descent method for the first 2500 steps and the conjugated gradient method for the next 2500 steps. Each system was then gradually raised from 0 K to 300 K in a 50 ps canonical ensemble (NVT) heating process. Finally, 50 ns production MD simulations were performed on the five systems in an isothermal isobaric ensemble (NPT) at a constant pressure (1 atm) and constant temperature (300 K) by applying the Langevin algorithm [39]. A cutoff equivalent to 10 Å was set for short-range electrostatics and van der Waals interactions. Long-range electrostatic interactions were processed using the particle mesh Ewald method [40] with cubic fourth-order B-spline interpolation and a 10^–5^ tolerance set for the direct sum tolerance. An integration step of 2 fs was set for the MD simulations. All covalent bonds involving hydrogen atoms were constrained at their equilibrium positions by the SHAKE method [41] with a tolerance of 10^–5^ Å.

All the MD trajectories were subsequently analyzed using PTRAJ module. The 1 ps interval saved coordinates were used to obtain the root-mean-square deviations (RMSDs), to calculate the change in distance between two atoms, and to analyze H bonds. The criteria for forming an H bond consists of an angle A-H-D larger than 120° and a distance between the acceptor atom and the donor atom smaller than 3.5 Å.

### Dynamic Cross-correlation Matrices

The Cα dynamic cross-correlation matrices (DCCM) were computed to reveal the correlative motions of proteins [42]. *C*(*i*,*j*) was calculated as follows:

$$C(i,j)=\frac{c(i,j)}{c{\left(i,i\right)}^{1/2}c{\left(j,j\right)}^{1/2}}$$

where *C*(*i*,*j*) is the covariance matrix element of the protein fluctuation between residues *i* and *j*.

The value of *C*(*i*,*j*) ranges from -1 to 1. Positive values suggest positively correlated movement (the same direction), whereas negative values suggest anticorrelated movement (the opposite direction).

## Results

### System Stabilities during MD Simulations

**Fig 2:**
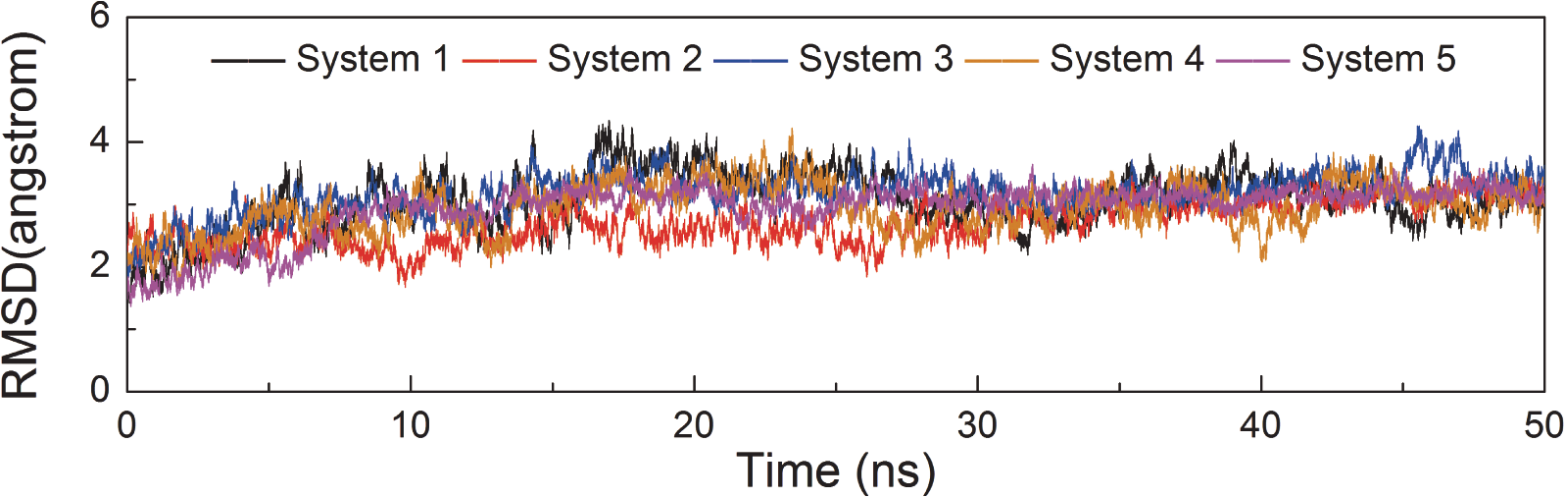
Time dependence of Cα atoms RMSDs of CDK9/cyclin T1 for five simulations in the 50 ns MD simulations. Systems 1, 2, 3, 4, and 5 are shown in black, red, blue, orange, and magenta, respectively. The same colors are maintained in the following Figs.

Conventional MD simulations of five systems, CDK9/cyclin T1/ATP/1MG complex (System 1), pCDK9/cyclin T1/ATP/1MG complex (System 2), CDK9/cyclin T1/ATP/2MG complex (System 3), pCDK9/cyclin T1/ATP/2MG complex (System 4), and pCDK9/cyclin T1/ATP/2MG/Tat complex (System 5), were performed in explicit water for 50 ns. The Cα atom RMSDs of the CDK9/cyclin T1 complexes in relation to the initial minimized structures as a function of simulation time for five systems were monitored. As shown in Fig 2, after approximately 10 ns of simulation, the RMSDs tended to converge in Systems 1, 2, 3, 4, and 5 with values of 3.07 ± 0.37 Å, 2.71 ± 0.30 Å, 3.18 ± 0.25 Å, 2.95 ± 0.31 Å, and 2.94 ± 0.30 Å, respectively. These values, along with the time dependence of total energies (data not shown), indicate that the five systems achieved a state of equilibrium and were sufficient for exploring the dynamic behavior of the studied systems.

### Salt Bridge Network in the pThr186 Binding Site

**Fig 3:**
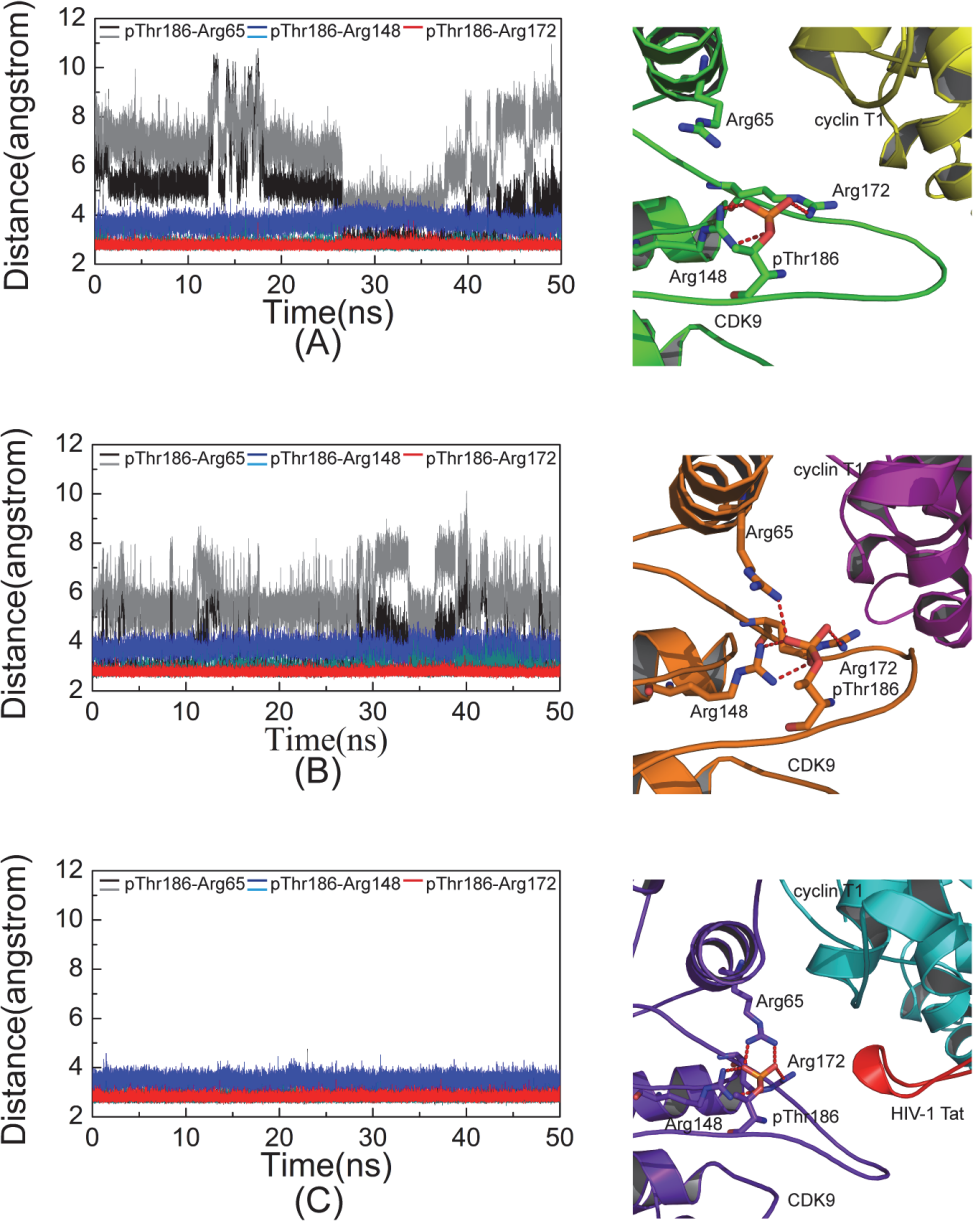
Distances between phosphate group of pThr186 and guanidine group of arginine triad versus simulation time. The distance between pThr186 and Arg65 are shown in black and gray, the distance between pThr186 and Arg148 are shown in dark cyan and blue, and the distance between pThr186 and Arg172 are represented as red in System 2 (A), System 4 (B), and System 5 (C). The salt bridges in the pThr186 binding site at the 50 ns snapshot in three corresponding systems are shown in the right panel. Residues involved in salt bridge formation (red dotted line) are described by the stick with a red oxygen atom, blue nitrogen atom, and orange phosphorus atom.

Thr186 at the active loop is phosphorylated in three constructed simulation systems (Systems 2, 4, and 5). A cluster of three arginine residues, including Arg65 at the αC helix, Arg148 at the catalytic loop, and Arg172 at the activation loop, constitutes the pThr186 binding site. System 5 contains HIV-1 Tat and features two salt bridges that were formed by Arg65 and Ary148 and one salt bridge that was formed by Arg172. Negatively charged pThr186 served as the hub to organize the positively charged triad. All five salt bridges were very stable throughout the simulation time (Fig 3C). By contrast, in Systems 2 and 4 without HIV-1 Tat binding, the resulting salt bridge networks were less stable. For example, in System 4, only one salt bridge was formed by Arg65, and it was less stable than that in System 5 (Fig 3B). In the System 2, the salt bridge formed between pThr186 and Arg65 was weakest (Fig 3A). Taken together, these data indicate that both Mg_1_^2+^ ion and HIV-1 Tat contributed to the stability of the salt bridge network formed by pThr186.

### Hydrogen Bonds in the ATP Binding Pocket

**Fig 4:**
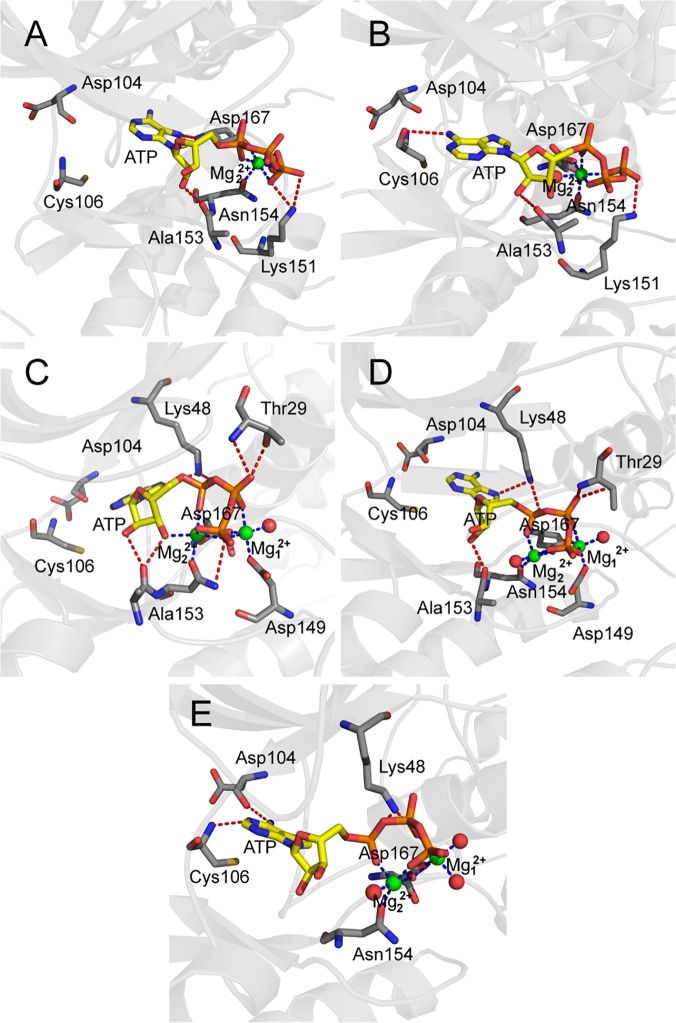
Hydrogen bonds and coordination bonds at the ATP binding pocket of CDK9 in five systems. CDK9 is shown as a gray ribbon with a gray stick representing residues involved in hydrogen bond or coordination bond. ATP is depicted by a yellow stick. All oxygen atoms, nitrogen atoms, and phosphate atoms are depicted in red, blue, and orange, respectively. Mg_1_^2+^ and Mg_2_^2+^ ions are exhibited as green spheres and water molecules are shown as red spheres. Red dotted lines indicate hydrogen bonds and blue dotted lines represent coordination bonds.

**Fig 5:**
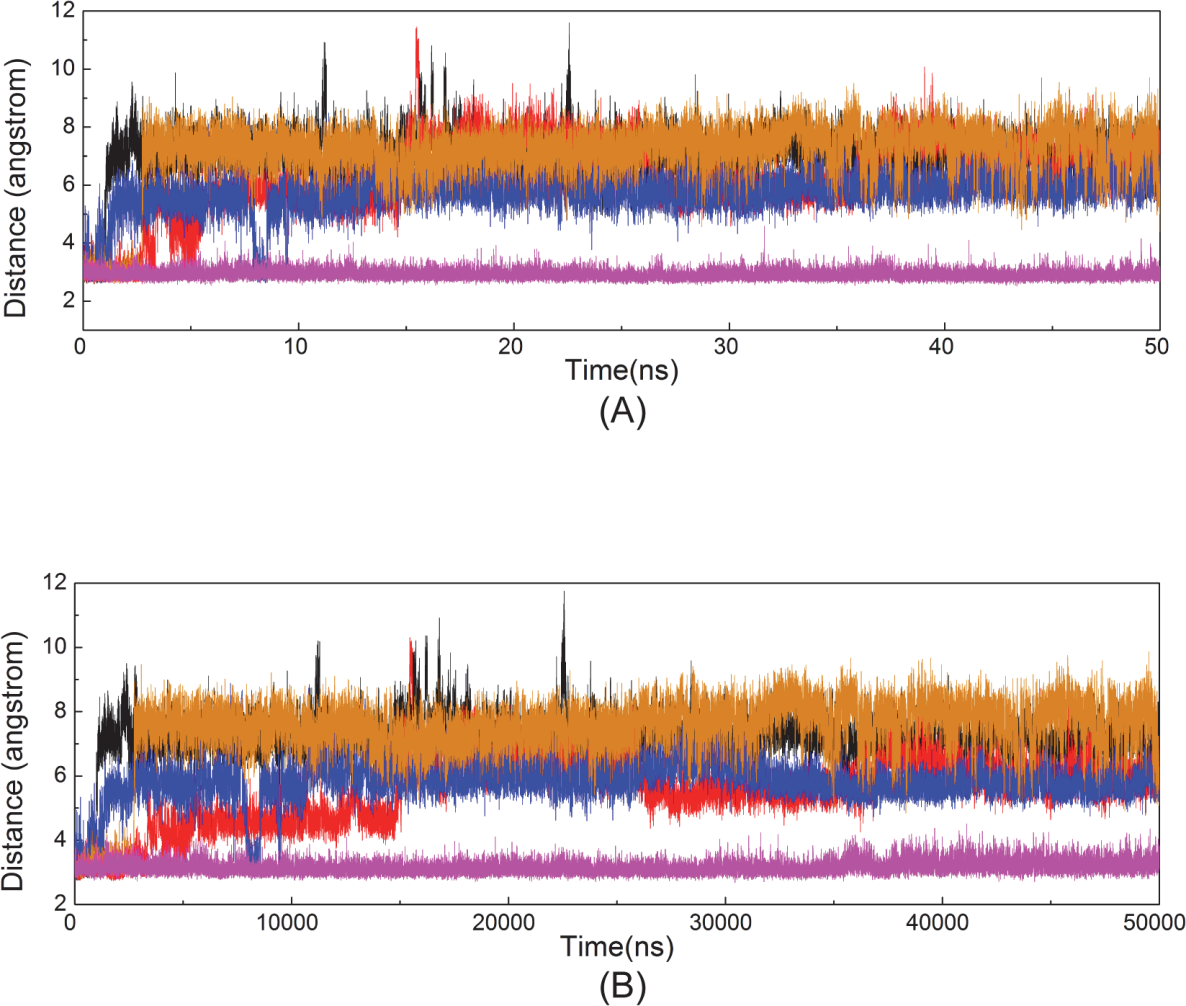
Time dependence of the distances between adenine of ATP and Asp104/Cys106 in the five simulations. (A) The distance between Asp104 carbonyl O atom and ATP N_6_ atom; (B) The distance between Cys106 amide N atom and ATP N_1_ atom.

The hydrogen bonds formed by ATP with CDK9 are shown in Fig 4 and tabulated in Table 2. Significant changes in ATP binding mode and ATP conformation were observed during simulation. System 5 showed that H bonds were formed between Asp104 backbone carbonyl O atom and ATP N_6_ atom, as well as between Cys106 backbone amide N atom and ATP N_1_ atom. As shown in Fig 5, these two H bonds were very stable in System 5 throughout simulation process. By contrast, the same H bonds were all broken in the other four systems. The breakage of these two H bonds resulted in the displacement of the adenine moiety of ATP from its original position. The occupancy values (%) of H bonds formed between the Asp104 carbonyl O atom and the ATP N_6_ atom were 2.01, 5.99, 3.19, and 5.30 in Systems 1, 2, 3, and 4, respectively, whereas those between the Cys106 amide N atom and the ATP N_1_ atom were 1.67, 5.40, 1.99, and 4.17, respectively. As shown in Fig 5, the rupture of the two H bonds between the adenine moiety of ATP and the hinge residues of CDK9 occurred at approximately 1 ns in nonphosphorylated Systems 1 and 3, and at approximately 2.7 ns in phosphorylated Systems 2 and 4. These observations suggest that the HIV-1 Tat is critical to the stabilization of H bonds between the adenine moiety of ATP and the hinge residues Asp104 and Cys106 of CDK9. In addition, another significant observation was related to the hydrogen bonding property of Lys48. In System 5, the Lys48 side chain N_ζ_ atom was within the hydrogen bonding distance of both the ATP O_α1_ and O_β2_ atoms. However, in the systems occupied by one Mg^2+^ (Systems 1 and 2), Lys48 was incapable of forming H bonds with ATP, whereas in the systems occupied by two Mg^2+^ ions (Systems 3 and 4), Lys48 formed one H bond with the ATP N_7_ atom and one H bond with the ATP O_α1_ atom. The pivotal H bond between ATP and invariant Lys48 (ATP-O_β2_…HZ-NZ-Lys48), which is crucial to the enzymatic catalysis reaction, was broken in all no HIV-1 Tat binding simulations (Table 2). This critical H bond causes the triphosphate moiety of ATP to assume the correct orientation during simulations. Although Lys48 was incapable of positioning its side chain in its optimal orientation in the two Mg^2+^ occupied systems (Systems 3 and 4), the presence of Mg_1_^2+^ ion contributed to the arrangement of Lys48 to obtain one correct H bond, that is, ATP-O_α1_…HZ-NZ-Lys48 interaction.

**Table 2:** Summary of the average distances and occupancies of hydrogen bonds between ATP and CDK9 in five 50 ns simulated systems.

Systems	Hydrogen bonds	Occupancy (%)	Distance (Å)
System1CDK9/cyclin T1/ATP/1MG	ATP-O_2*_-H_2'_…O-Ala153	45.56	2.846±0.21
	ATP-N_7_…H-N-Asp167	37.95	3.268±0.15
	ATP-O_γ1_…HZ-NZ-Lys151	70.38	3.025±0.23
	ATP-O_γ2_…HZ-NZ-Lys151	87.12	2.880±0.18
System2 pCDK9/cyclin T1/ATP/1MG	ATP-O_2*_-H_2'_…O-Ala153	93.76	2.753±0.15
	ATP-N6-H60…O-Cys106	63.18	2.990±0.18
	ATP- O_γ1_…HZ-NZ-Lys151	100.00	2.716±0.08
System3 CDK9/cyclin T1/ATP/2MG	ATP-O_2*_-H_2'_…O-Ala153	67.47	2.798±0.16
	ATP-O_3*_-H_3'_…O-Ala153	54.33	2.980±0.21
	ATP-O_γ3_…H21-ND2-Asn154	73.28	3.097±0.16
	ATP-O_β1_…HG1-OG1-Thr29	95.43	2.645±0.11
	ATP-O_β1_…H-N-Thr29	92.77	2.949±0.16
	ATP-N_7_…HZ-NZ-Lys48	86.94	3.072±0.16
	ATP-O_α1_…HZ-NZ-Lys48	99.54	2.719±0.08
	ATP-O_γ1_…HZ-NZ-Lys151	63.34	2.762±0.11
System4 pCDK9/cyclin T1/ATP/2MG	ATP-O_2*_-H_2'_…O-Ala153	81.63	2.797±0.17
	ATP-O_β1_…H-N-Thr29	98.02	2.816±0.10
	ATP-O_β1_…HG1-OG1-Thr29	98.07	2.738±0.16
	ATP-N_7_…H-N-Asp167	60.15	3.254±0.15
	ATP-N_7_…HZ3-NZ-Lys48	93.01	2.982±0.14
	ATP-O_α1_…HZ2-NZ-Lys48	97.12	2.707±0.08
System5 pCDK9/cyclin T1 /ATP/ 2MG/Tat	ATP-N_6_-H_60_…O-Asp104	99.16	2.915±0.15
	ATP-N_1_…H-N-Cys106	89.26	3.117±0.15
	ATP-O_α1_…HZ3-NZ-Lys48	99.83	2.711±0.08
	ATP-O_β2_…HZ2-NZ-Lys48	80.51	2.973±0.16

### ATP Conformation in the ATP Binding Pocket

**Fig 6:**
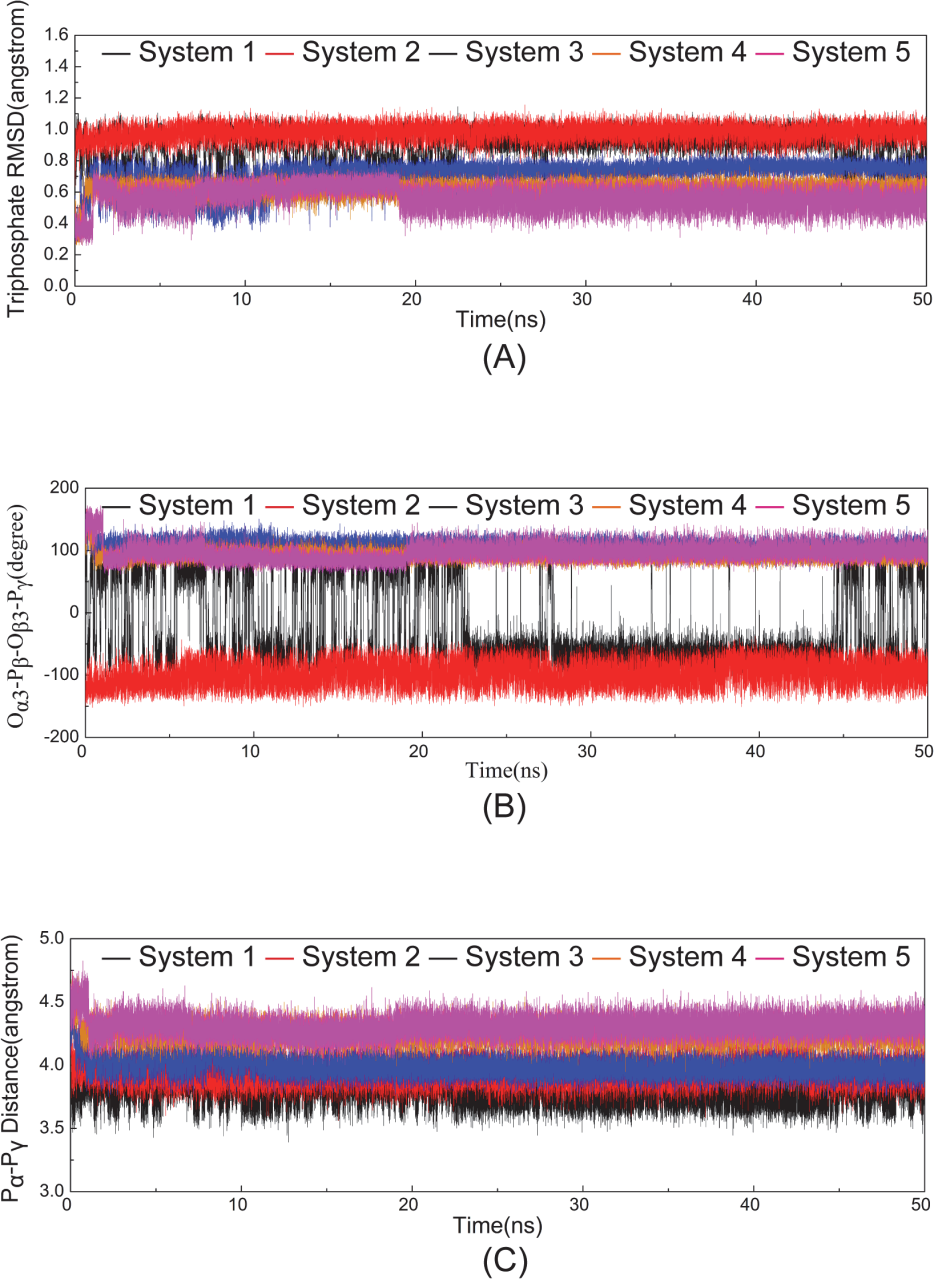
Time dependence of RMSDs of ATP in five systems versus simulation time in the five systems. (A) The triphophate moiety of ATP. (B) The dihedral O_α3_-P_β_-O_β3_-P_γ_. (C) The P_α_ and P_γ_ atoms of triphosphate moiety of ATP.

Apart from the differences in the formation of H bonds between ATP and CDK9 in various systems, the simulations also indicated that the binding of both the Mg_1_^2+^ ion and HIV-1 Tat significantly reduced the magnitude of nanosecond timescale fluctuations in ATP phosphates. The heavy atom RMSDs of ATP triphosphate moiety in the five systems in relation to its conformation in the initial minimized structure were monitored and calculated to be 0.89 ± 0.09 Å, 0.98 ± 0.04 Å, 0.72 ± 0.05 Å, 0.62 ± 0.04 Å, and 0.56 ± 0.06 Å for Systems 1, 2, 3, 4, and 5, respectively (Fig 6A). The average RMSD values in the first two systems with one Mg^2+^ ion binding were higher than those in the remaining three systems with the binding of two Mg^2+^ ions, which is consistent with the observed reorientation of the triphosphate moiety in Systems 1 and 2. With the Mg_1_^2+^ ion at the binding pocket in Systems 3, 4, and 5, the additional magnesium-mediated ionic interactions reduced the conformation fluctuation of the ATP phosphates. In addition, HIV-1 Tat binding further reduced the triphosphate RMSD values. We also analyzed the conformational change in the triphosphate moiety of ATP by monitoring the dihedral O_α3_-P_β_-O_β3_-P_γ_ and the distance between the P_α_ and P_γ_ atoms in the five simulation systems as a function of time (Fig 6B and 6C). The values of dihedral O_α3_-P_β_-O_β3_-P_γ_ in System 1 significantly fluctuated during simulation but were rather stable in the other four systems (‒98.56 ± 17.02°, 106.74 ± 7.86°, 91.84 ± 6.52°, and 97.21 ± 10.80° for Systems 2, 3, 4, and 5, respectively). The distances between the P_α_ and P_γ_ atoms were 3.78 ± 0.08 Å, 3.94 ± 0.07 Å, 3.99 ± 0.06 Å, 4.24 ± 0.06 Å, and 4.31 ± 0.06 Å in Systems 1, 2, 3, 4, and 5, respectively. In Systems 1 and 2, which lacked the Mg_1_^2+^ ion, the dihedral angle O_α3_-P_β_-O_β3_-P_γ_ and the distance between the two phosphorus atoms (P_α_ and P_γ_) were significantly different from those observed in the binding systems with two Mg^2+^ ions.

### Magnesium–Ligand Coordination

**Fig 7:**
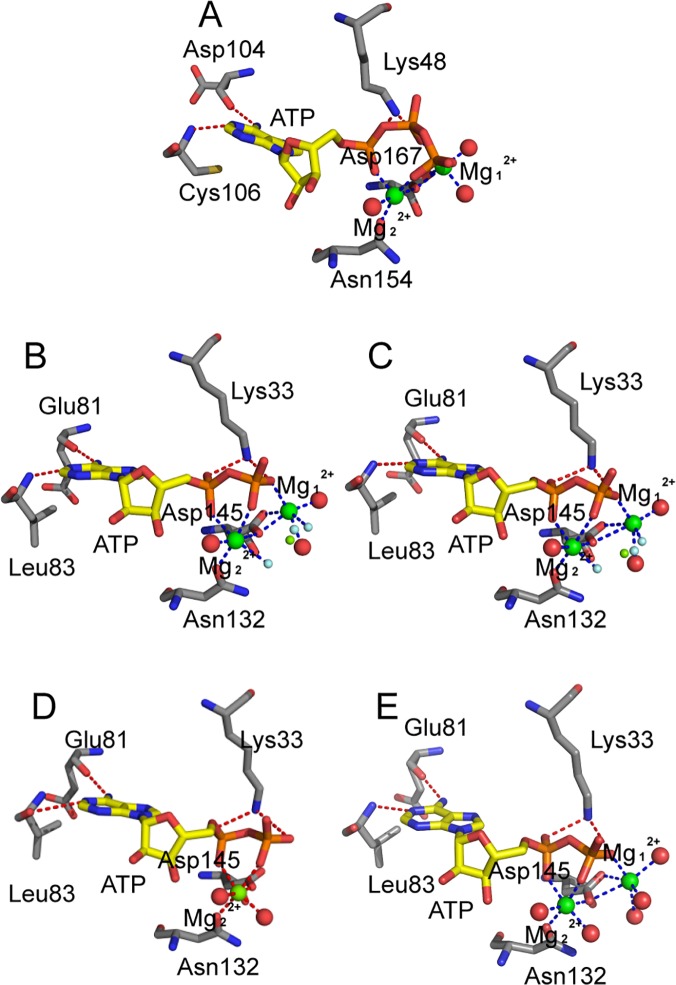
Schematic representation of the hydrogen bonds and coordination bonds in ATP active site in System 5 and in four CDK2 X-ray structures. (A) pCDK9/cyclin T1/ATP/2MG/Tat complex in System 5, (B) TS complex pCDK2/cyclinA/ADP/2MG/MgF_3_^-^/peptide (PDB code 3QHR) solved at pH 8.0, (C) TS complex pCDK2/cyclinA/ADP/2MG/MgF_3_^-^/peptide (PDB code 3QHW) solved at pH 8.25, (D) pCDK2/cyclinA/ADP/1MG complex (PDB code 4II5), (E) pCDK2/cyclinA/ADP/2MG complex (PDB code 4I3Z).

To analyze the magnesium–ligand coordination mode in our simulations, we compared our simulation results with the X-ray crystal structures of CDK2. Yong *et al*. recently obtained several ADP cocrystallized pCDK2/cyclin A structures [21,22]. Two of these structures comprise pCDK2/cyclin A bound to ADP, substrate peptide, and trigonal-planar MgF_3_^-^ ion, a mimic for the γ-phosphate of ATP (PDB code 3QHR and 3QHW), which are very similar to the TS complexes. Two crystal structures (Fig 7B and 7C) showed Mg_1_^2+^ ions that were coordinated by one of the MgF_3_^-^ fluorine atoms, an ADP β-phosphate oxygen atom, two side chain carboxyl oxygen atoms of Asp145 at the DFG motif, and oxygen atoms of two water molecules. Mg_2_^2+^ ion also formed six coordination bonds with one of the MgF_3_^-^ fluorine atoms, ADP α- and β-phosphate oxygen atoms, one carboxyl oxygen atom of Asp145, one side chain carbonyl oxygen atom of Asn132, and one oxygen atom of water molecule. Each Mg^2+^ ion maintained a hexa-coordinated octahedral geometry, which was also observed in the pCDK2/cyclin A complex bound to ADP with one Mg^2+^ ion (PDB code 4II5) or with two Mg^2+^ ions (PDB code 4I3Z), as shown in Fig 7D and 7E, respectively. The difference is that the coordinated MgF_3_^-^ fluorine atom in the 3QHR and 3QHW is replaced by the oxygen atom of water molecule in the 4II5 and 4I3Z.

In simulation System 5 (Fig 7A), the Mg_1_^2+^ ion also formed six coordination bonds with ATP β- and γ-phosphate oxygen atoms, two side chain carboxyl oxygen atoms of invariant Asp167 (Asp145 in CDK2) of the DFG motif, and two oxygen atoms of water molecules. Compared with Mg_1_^2+^ ion in the two CDK2 TS complex X-ray structures, five coordination bonds were found to be similar, and only one coordination bond was different, that is, the γ-phosphate oxygen acted as a coordinating group in place of fluorine in MgF_3_^-^.

An Mg_2_^2+^ ion also exhibits hexa-coordinated octahedral geometry, but only five coordination bonds were observed during simulation. Mg_2_^2+^ was coordinated to ATP α- and γ-phosphate oxygen atoms, a carboxyl oxygen atom of Asp167 (Asp145 in CDK2), a side chain carbonyl oxygen of Asn154 (Asn132 in CDK2), and an oxygen atom in water. In this work, the γ-phosphate oxygen, instead of β-phosphate oxygen, is a coordinating group, and an unoccupied coordination position is observed. This position is occupied by fluorine ion in MgF_3_^-^ in the CDK2 TS complex.

All three solvent water molecules involved in the coordination at the ATP binding pocket rapidly reached their equilibrium positions at the beginning of the simulation in System 5 and remained at their equilibrium positions at the end of 50 ns simulation, but no water molecule filled the unoccupied coordination position. This condition may be attributed to the fact that the unoccupied position is required for CDK9 to achieve its TS. A possible scenario is that the β-phosphate oxygen atom will form a coordination bond with Mg_2_^2+^, whereas the cleaved γ-phosphate oxygen moves into the unoccupied coordination position to form another coordination bond.

In binding simulation systems with two Mg^2+^ ions (Systems 3, 4, and 5), the second Mg^2+^ ion (Mg_1_^2+^) formed six coordination bonds, similar to that observed in the three CDK2 crystal structures (Fig 7). Lu *et al*. [43,44] showed that the hexa-coordinated octahedral geometry of Mg^2+^ ion at site 1 was important for GSK3β activity, and dislodging this natural cofactor Mg^2+^ ion by a nonnative Ca^2+^ ion, which preferred a hepta-coordinated geometry, eliminated enzymatic activity.

### Critical Conformational Change

**Fig 8:**
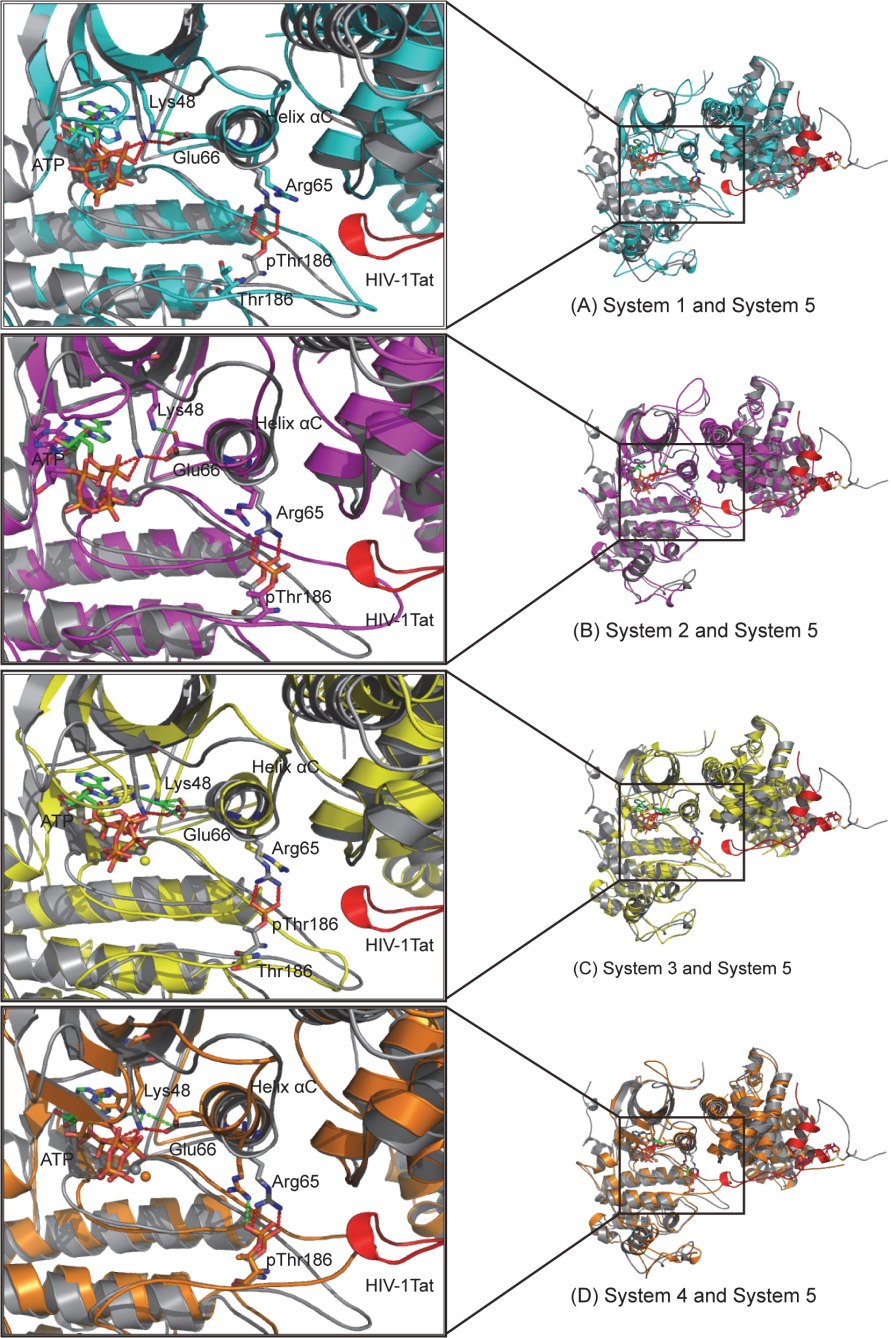
Superimposition of the CDK9 in snapshot at 50 ns in System 5 with those in the other four systems. (A) Superimposition of the CDK9 in System 1 (cyan) and System 5; (B) superimposition of the CDK9 in System 2 (magenta) and System 5; (C) superimposition of the CDK9 in System 3 (yellow) and System 5; (D) superimposition of the CDK9 in System 4 (orange) and System 5. The pCDK9/cyclin T1 is gray and HIV-1 Tat is red in System 5. The hydrogen bonds or salt bridges formed in System 5 are depicted by the red dotted line, and these interactions in other systems are described by the green dotted line.

We superimposed the CDK9 subunit in pCDK9/cyclin T1/ATP/2Mg/Tat complex of System 5 with the CDK9 subunit in the other four systems to explore the critical conformation change of CDK9, which results in the nonproductive binding of ATP. Upon closer inspection, the prominent helix αC, a key structural element, was shifted in non-HIV-1 Tat binding systems. As shown in Fig 8D, the unoccupied space above the activation loop in System 4 caused the activation loop to rotate upward, followed by the upward shift in the helix αC. As a consequence of this conformational change, the position of two conserved hydrogen-bond interacting residues (Glu66 and Lys48) that are important for the correct localization of ATP triphosphate were relocated, which consequently caused Lys48 to form different H bonds with ATP. Systems 3 and 4 showed that Lys48 H bonds with O_α1_ and N_7_ atoms instead of forming H bonds with O_α1_ and O_β2_ atoms, as is the case in System 5. In the one Mg^2+^ binding systems, the location and conformation of ATP were altered to a greater degree than those in the two Mg^2+^ binding systems. The absence of Mg_1_^2+^, ATP β-phosphate, and γ-phosphate oxygen atoms in Systems 1 and 2 were rotated and displaced, which were also demonstrated by the significant differences in RMSDs of the triphosphate moieties and the dihedral angles O_α3_-P_β_-O_β3_-P_γ_. In System 1, the helix αC moved forward instead of exhibiting the upward shift observed in the other three systems. The side chain of Arg65 was curled in the nonphosphorylated systems, possibly because of charge repulsion.

## Discussion

Phosphorylation of the activation loop is a prerequisite for kinase activation [45,46]. The ion pairs between Arg65 and pThr186 in CDK9, between Arg50 and pThr160 in CDK2, and between His87 and pThr197 in catalytic subunit of PKA (PKAc) [47], which connect the activation loop of the C terminal lobe and helix αC of the N terminal lobe, are key structural elements of active kinases. A more stable salt bridge network between pThr186 and the arginine triad was formed in the presence of the HIV-1 Tat binding system. Notably, cyclin T1 in the first four systems was based on one crystal structure (PDB code 3BLQ) [8], which included three point mutations at residues Q77R, E96G, and F241L. By contrast, cyclin T1 in the last system is based on a different crystal structure (PDB code 3MIA) [9] and is a wild-type protein. Among the three mutations, E96G is located at the CDK/cyclin interface, and Glu96_T1_ was found to form salt bridge interactions with Arg65_CDK9_ in System 5. To investigate the function of Glu96_T1_ in the formation of Arg65–pThr186 ion pairs, we checked the twenty CDK9/cyclin T1 crystal structures in the PDB database. Only seven of them contained wild-type Glu96 in cyclin T1. The crystal structures of seven CDK9/cyclin T1 complexes are listed in S1 Table and the salt bridge network formed by Glu96_T1_, Arg65_CDK9_ and pThr186_CDK9_ are shown in S1 Fig. Two Arg65–pThr186 ion pairs were observed in the four crystal structures with Tat binding (PDB code 3MIA [9], 3MI9 [9], 4OGR [17], and 4OR5 [18]). The CDK9/cyclin T1 complex binding to AFF4 scaffold (PDB code 4IMY [11]) showed the existence of an Arg65–pThr186 ion pair. In other non-Tat binding crystal structures, only one Arg65–pThr186 ion pair was found in the crystal structure (PDB code 3TNH [10]), and no ion pair was detected in the other crystal structure (PDB code 3TNI [10]). These data from crystal structures are consistent with our MD simulation results, which suggested that only one less stable Arg65–pThr186 salt bridge was formed in the complex owing to the absence of Tat binding. Schulze-Gahmen *et al*. [11] have recently found that although HIV-1 Tat increases P-TEFb affinity for AFF4 scaffold, the structure of CDK9 kinase subunit structure did not show significant change upon AFF4 binding to P-TEFb and further addition of AFF_2–73_ did not stimulate the kinase activity of Tat-P-TEFb complex. Taken together, these results demonstrate that the Glu96 in the cyclin T1 may contribute to the reorientation of the side chain of Arg65, but does not significantly affect the interaction between Arg65 and pThr186. Therefore, HIV-1 Tat binding contributed to the formation of a stable salt bridge network.

Although the conformation differences between the structures of pCDK9/cyclin T1/ATP/1MG complex (PDB code 3BLQ) and pCDK9/cyclin T1/AMP-PNP/1MG/Tat complex (PDB code 3MIA) were minor (RMSD = 1.220 Å), significant differences in dynamic behavior were observed in the last two systems. Both the wild-type and triple mutant complexes have the same *K*_Mapp'ATP_ value determined by Baumli *et al*. [10], which indicated that ATP binding was unaffected by mutation. Therefore, the different dynamic behavior of Systems 4 and 5 can be attributed to HIV-1 Tat binding. Repositioning of the helix αC is widely exploited to explore the modulation of protein kinase activities. In this work, the shift of helix αC caused Glu66 and its partner Lys48 to assume incorrect positions, thereby leading to the disruption of H bonds between Lys48 N_ζ_ atom and ATP O_β2_ atom.

The presence of Mg_1_^2+^, along with regulator HIV-1 Tat binding, accounted for the extremely similar architecture in CDK9 as that observed in some other kinase crystal structures containing two metal ions and nonhydrolyzable ATP analog (or ADP). These structures include pCDK2/cyclin A/ADP/2MG/MgF_3_^-^/peptide complex (PDB code 3QHR [21]), GSK3β/AMP-PNP/2MG complex (PDB code 1PYX [30]), MST3/ADP/2MN complex (PDB code 3A7J [48]), PKAc/AMP-PCP/2MG complex (PDB code 4IAC [49]), PKB/AMP-PNP/2MN complex (PDB code 1O6L [50]) and p38γ/AMP-PNP/2MG complex(PDB code 1CM8 [51]) (S2 Table). Schematic representation of the H bonds and coordination bonds in the ATP active site of these kinase crystal structures are shown in S2 Fig. All share similar binding mode between kinase and ADP or ATP analog as that found in System 5. Adenine forms two H bonds with two residues in hinge region. The Lys interacts with α- and β-phosphate oxygen atoms, and residues Asn and Asp coordinate to metal ions.

Many enzymes catalyze similar reactions that release the γ-phosphate from a nucleotide triphosphate (NTP), but the number of catalytic metals is not always conserved. Myosin [52–53], elongation factor Tu [54] and TIP49 AAA+ ATPase [55] have different ATP binding mode and ATP hydrolysis mechanism is involved in a single Mg^2+^ ion. Also, there are two metal ions captured in several crystal structures of catalytic subunit of cAMP-dependent kinase (PKAc), including the reactant, PKAc/ATP/2MG complex (PDB code 4IAC [49]), the product, PKAc/ADP/2MG complex (PDB code 4IAD, 4IAF [49]), and the transition state analog, PKAc/ADP/AlF_3_/substrate peptide/2MG complex (PDB code 1L3R [56]). In the transition state of PKAc (S3A Fig), the arrangement of the side chain oxygen atom of Ser in the substrate peptide (OγSer-P), Al^3+^ ion and the oxygen atom of β phosphate is in line. In PKAc, the transition states of the phosphoryl-transfer reaction have been classified as associative or dissociative transition states. In the associative mechanism, one of the oxygen atoms of the γ phosphate group to be transferred acts as a base, accepting the proton initially attached to OγSer-P [57]. In the dissociative mechanism, Asp166 residue serves as a catalytic base that accepts substrate peptide proton during the phosphorylation process [58–60]. Although there are two disputed phosphoryl-transfer mechanisms, OγSer-P has to attack ATP γ phosphorus in S_N_2-like reaction, producing the direct displacement of the ADP moiety, without the involvement of any solvent water. Alignment of the MD snapshot of System 5 with PKAc transition state analog is shown in S3C Fig. With the engagement of both HIV-1 Tat and Mg_1_^2+^, CDK9 in the System 5 established the catalytically-competent conformation as observed in the PKAc. Based on the similar ATP binding mode and ATP catalytically-competent conformation obtained from the System 5, CDK9 may share a similar mechanism of phosphoryl-transfer reaction with its homologous protein PKAc.

The triphosphate moiety binding subpocket is extremely electronegative and includes a number of conserved negative charges, such as Glu66 (Glu51 in CDK2), Glu149 (Glu127 in CDK2), and Asp167 (Asp145 in CDK2). The two Mg^2+^ ions are utilized to accommodate the phosphates into the active site environment. We modeled the second Mg^2+^ ion (Mg_1_^2+^ ion at site 1) in MD simulations to explore its functional role. Significant differences were found between one Mg^2+^ ion binding simulations and binding simulations of two Mg^2+^ ions in the RMSDs of triphosphate moieties of ATP, the dihedral O_α3_-P_β_-O_β3_-P_γ_, and the distance between the P_α_ and P_γ_ atoms. Owing to the loss of Mg_1_^2+^ mediated interaction with CDK9, the triphosphate moiety was rotated and shifted in one Mg^2+^ ion binding simulations. Szarek *et al*. [61] used the methodology of differential transition state stabilization to investigate the phosphoryl transfer reaction catalyzed by PKAc. They indicated that Mg2 (labeled Mg_2_^2+^ in our MD simulation) and Mg1 (labeled Mg_1_^2+^ in our MD simulation) contributed -32.36 kcal/mol and -15.15 kcal/mol to stabilize the transition state of PKAc, respectively. Zhao *et al*. [21] found that Mg^2+^ concentration could represent an important regulator of CDK2 activity in vivo. Furthermore, both of their data demonstrated that binding of the second Mg^2+^ ion rendered ATP in more ordered conformation and in additional interactions with the protein. The correct orientation and conformation of ATP triphosphate moiety is crucial to the phosphoryl-transfer reaction between ATP γ-phosphate and the threonine hydroxyl group on the substrate, which has been corroborated by experimental evidence and computational studies [62–68]. Our MD simulation study also provided convincing evidence that both Mg^2+^ ions are needed for CDK9 to recruit ATP and localize the triphosphate moiety of ATP in the correct position and conformation.

**Fig 9:**
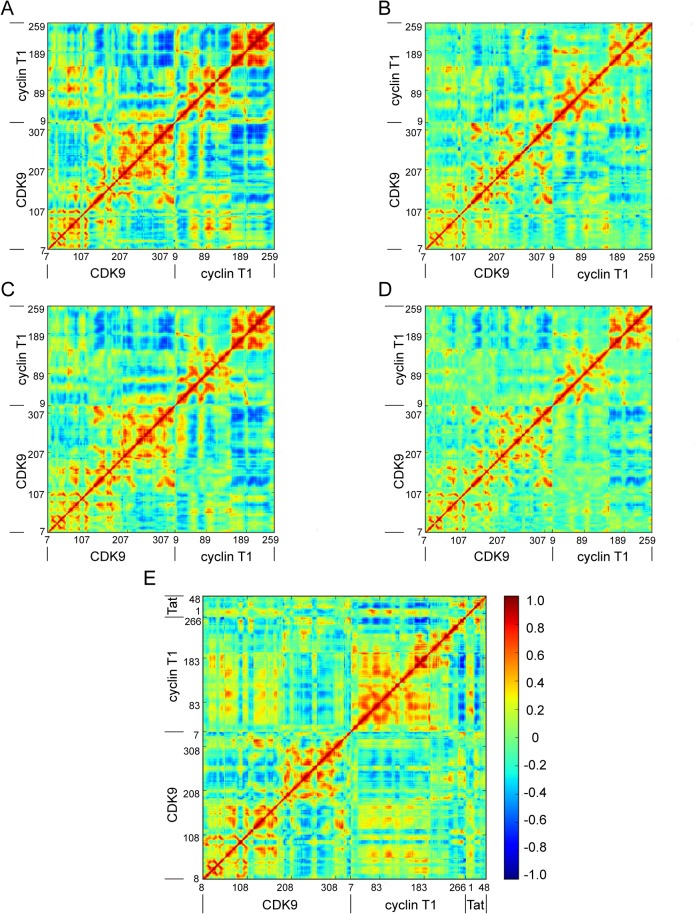
DCCM for System 1 (A), System 2 (B), System 3 (C), System 4 (D), and System 5 (E).

The DCCM for the five systems was further analyzed to determine the effect of the phosphorylated state of CDK9, Mg^2+^ ions, and HIV-1 Tat on the conformational motions of complexes. Inspection of the nonphosphorylated states of CDK9 (Fig 9A and 9C) and the phosphorylated states of CDK9 (Fig 9B, 9D and 9E) revealed that the nonphosphorylated states of CDK9 displayed stronger anticorrelated motions than the phosphorylated states of CDK9. These results indicate that phosphorylation of CDK9 has the potential to stabilize the conformational plasticity of CDK9. A comparison between the one Mg^2+^ ion binding (Fig 9A) and the two Mg^2+^ ion binding systems (Fig 9C) showed that the two Mg^2+^ ion binding system significantly reduced the conformational motions relative to the one Mg^2+^ ion binding system. However, this effect was not obviously observed in the phosphorylated states of CDK9 with one Mg^2+^ ion binding (Fig 9B) and two Mg^2+^ ion binding systems (Fig 9D). In addition, when compared with to the structure without HIV-1 Tat (Fig 9D), binding of HIV-1 Tat to the CDK9/cyclin T1 complex reduced the conformational motions of complex (Fig 9E). Cumulatively, these data suggest that the phosphorylated state of CDK9, the second Mg^2+^ ion, and HIV-1 Tat binding are capable of stabilizing the conformational flexibility of complexes.

## Conclusions

The present MD simulations provided valuable insight into the functional roles of regulator HIV-1 Tat and a second Mg^2+^ ion at site 1. HIV-1 Tat binding was is important to stabilize the salt bridge network in the pThr186 binding site. HIV-1 Tat binding also occupied the space above the activation loop, hence blocking upward shift of the activation loop. The strong Arg65–pThr186 ion pair and steric hindrance fixed the prominent helix αC in its appropriate position, which consequently resulted in the correct location of the highly conserved glutamate residue Glu66 and its partner Lys48. The second Mg^2+^ ion at site 1 was required for CDK9 to realize its function. The ion had a crucial role to fix the triphosphate moiety in its appropriate position by establishing coordination bonds with β- and γ-phosphate oxygen atoms. HIV-1 Tat binding, along with the appearance of two Mg^2+^ ions, resulted in an optimized magnesium-ligand coordination mode and the reproduction of the active site architectural characteristics in phosphorylated CDK9. The ATP binding mode in CDK9, which involves H bonds between the adenine moiety of ATP and hinge region residues, H bonds formed by the conserved residue Lys48 with the O_α1_ and O_β2_ atoms of ATP, and the hexa-coordinated octahedral geometry of Mg^2+^ with conserved residues Asp167 (Asp145) and Asn154 (Asn132), is very similar to the conformation captured in crystal structures of the pCDK2/cyclin A TS complex. All these results provide significant insight into the CDK activation/regulation processes and might help with the design of P-TEFb inhibitors to target HIV-1 transcription.

## Supporting Information

S1 Fig The salt bridge network formed by Glu96_T1_, Arg65_CDK9_ and pThr186_CDK9_ in 7 crystal structures of CDK9/cyclin T1 complex. (A) pCDK9/cyclin T1/Tat/AMP-PNP complex (PDB code 3MIA) (B) pCDK9/cyclin T1/Tat complex (PDB ID 3MI9), (C) pCDK9/cyclin T1/Tat/AFF4/adenosine complex (PDB code 4OGR), (D) pCDK9/cyclin T1/Tat/AFF4 complex (PDB code 4OR5), (E) pCDK9/cyclin T1/AFF4/AMP complex (PDB code 4IMY), (F) pCDK9/cyclin T1/CAN508 complex(PDB code 3TNH) and (G) pCDK9/cyclin T1 complex (PDB code 3TNI). CDK9, cyclin T1 and HIV-1 Tat are shown in grey, magenta and red ribbon, respectively. The Arg65, pThr186 and Glu96 are drawn as sticks. The salt bridges are shown as red dotted lines.
(TIF)

S2 Fig Schematic representation of the hydrogen bonds and coordination bonds in ATP active site in kinase crystal structures containing 2 metal ions and ATP analog. (A) pCDK2/cyclin A/ADP/2MG/MgF_3_^-^/peptide complex (PDB code 3QHR), (B) GSK3β/AMP-PNP/2MG complex (PDB code 1PYX), (C) MST3/ADP/2MN complex (PDB code 3A7J), (D) PKAc/AMP-PCP/2MG complex (PDB code 4IAC), (E) PKB/AMP-PNP/2MN complex (PDB code 1O6L), (F) p38γ/AMP-PNP/2MG complex (PDB code 1CM8).
(TIF)

S3 Fig Alignment of MD snapshot of System 5 with PKAc transition state analog. (A) PKAc/ADP/AlF_3_/substrate peptide/2MG complex (PDB code 1L3R), (B) MD snapshot of System 5, (C) the alignment of MD snapshot of System 5 with PKAc transition state analog.
(TIF)

S1 Table The compositions of 7 crystal structures of CDK9/cyclin T1 complex. (DOCX)

S2 Table Kinase crystal structures containing 2 metal ions and ATP analog. (DOCX)
