# Supplementary figures and images for: Determining the Functions of HIV-1 Tat and a Second Magnesium Ion in the CDK9/Cyclin T1 Complex: A Molecular Dynamics Simulation Study
Source: PLoS One. 2015 Apr 24;10(4):e0124673. doi: 10.1371/journal.pone.0124673 (PMC4409394; doi:10.1371/journal.pone.0124673)

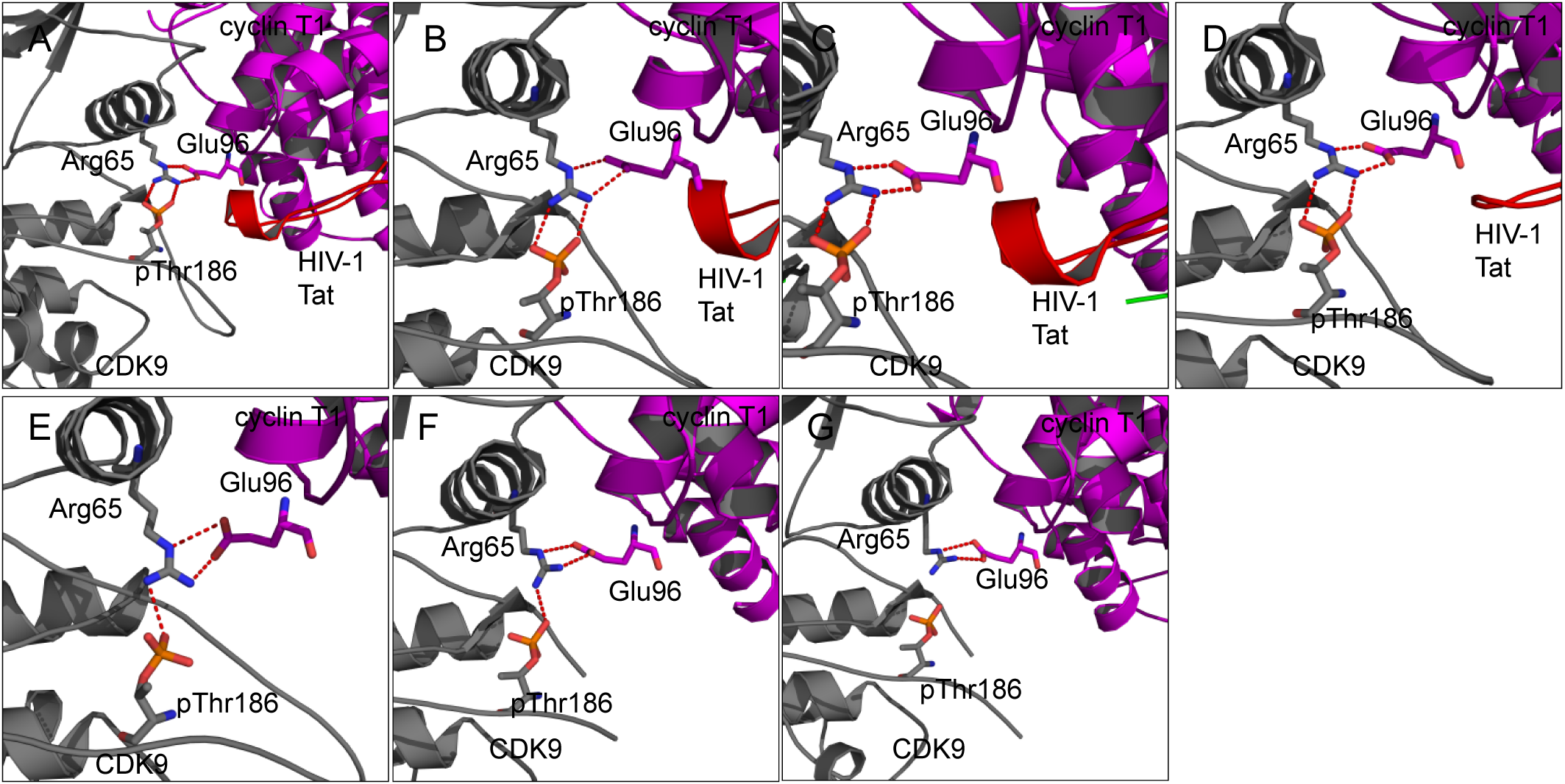

Supplement: S1 Fig — (A) pCDK9/cyclin T1/Tat/AMP-PNP complex (PDB code 3MIA) (B) pCDK9/cyclin T1/Tat complex (PDB ID 3MI9), (C) pCDK9/cyclin T1/Tat/AFF4/adenosine complex (PDB code 4OGR), (D) pCDK9/cyclin T1/Tat/AFF4 complex (PDB code 4OR5), (E) pCDK9/cyclin T1/AFF4/AMP complex (PDB code 4IMY), (F) pCDK9/cyclin T1/CAN508 complex(PDB code 3TNH) and (G) pCDK9/cyclin T1 complex (PDB code 3TNI). CDK9, cyclin T1 and HIV-1 Tat are shown in grey, magenta and red ribbon, respectively. The Arg65, pThr186 and Glu96 are drawn as sticks. The salt bridges are shown as red dotted lines. (TIF) [file pone.0124673.s001.tif]

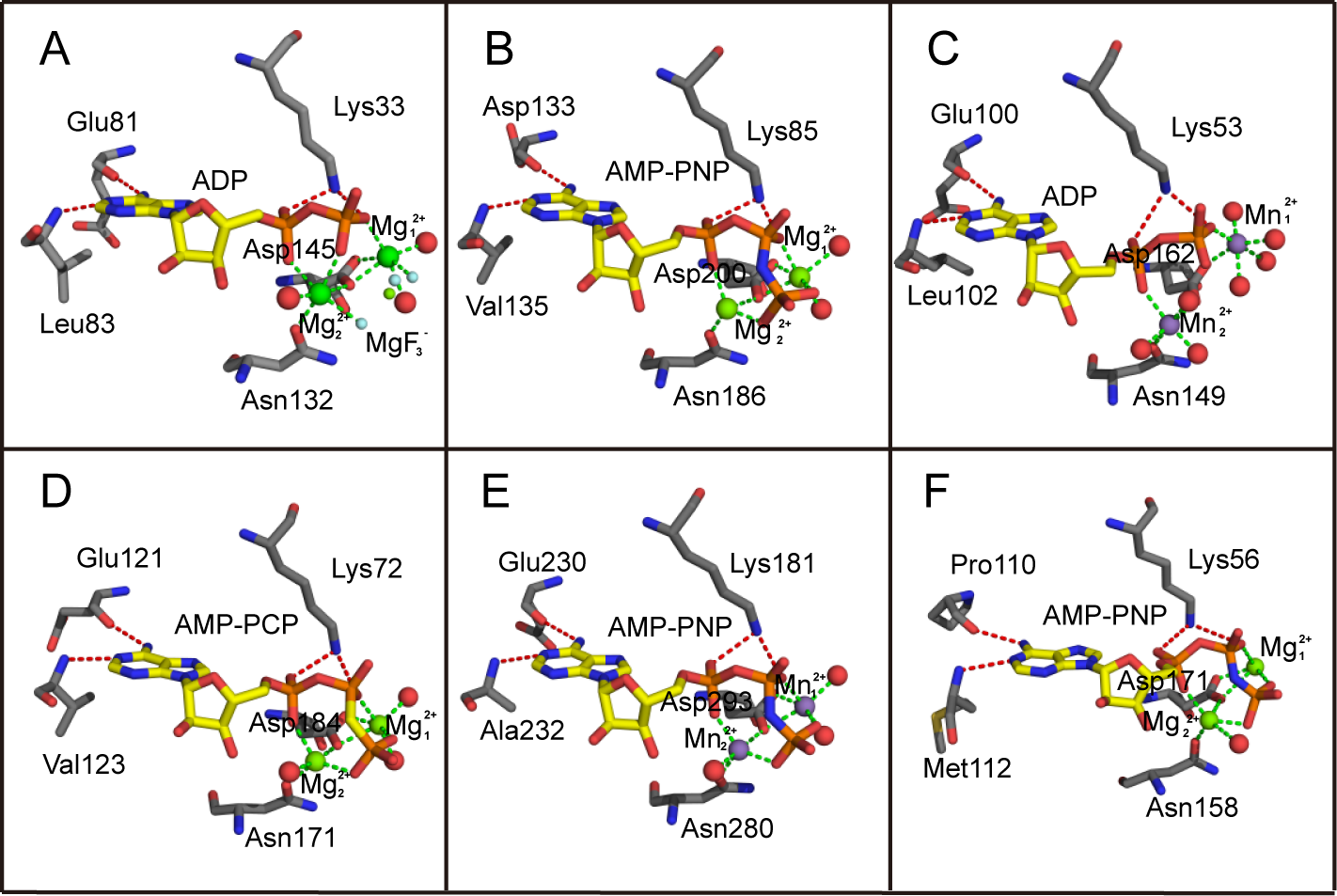

Supplement: S2 Fig — (A) pCDK2/cyclin A/ADP/2MG/MgF3 -/peptide complex (PDB code 3QHR), (B) GSK3β/AMP-PNP/2MG complex (PDB code 1PYX), (C) MST3/ADP/2MN complex (PDB code 3A7J), (D) PKAc/AMP-PCP/2MG complex (PDB code 4IAC), (E) PKB/AMP-PNP/2MN complex (PDB code 1O6L), (F) p38γ/AMP-PNP/2MG complex (PDB code 1CM8). (TIF) [file pone.0124673.s002.tif]

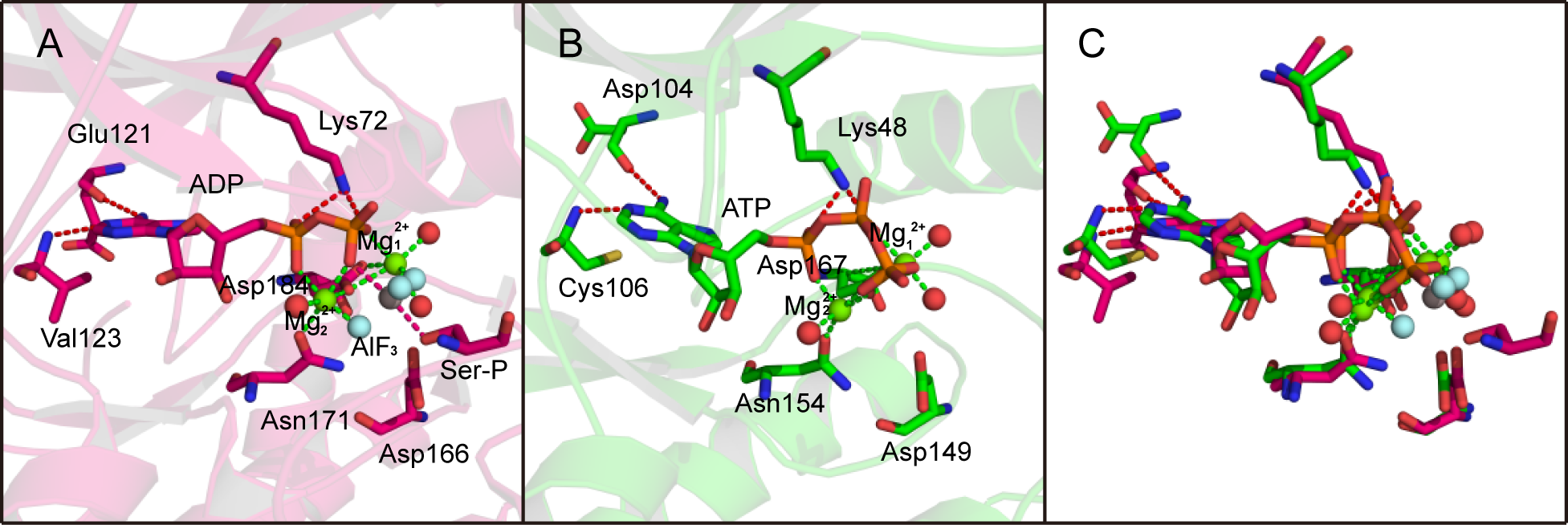

Supplement: S3 Fig — (A) PKAc/ADP/AlF3/substrate peptide/2MG complex (PDB code 1L3R), (B) MD snapshot of System 5, (C) the alignment of MD snapshot of System 5 with PKAc transition state analog. (TIF) [file pone.0124673.s003.tif]
