# Supplementary material for: Determining the Functions of HIV-1 Tat and a Second Magnesium Ion in the CDK9/Cyclin T1 Complex: A Molecular Dynamics Simulation Study
Source: PLoS One. 2015 Apr 24;10(4):e0124673. doi: 10.1371/journal.pone.0124673 (PMC4409394; doi:10.1371/journal.pone.0124673)
Supplement: S1 Table — (DOCX) [file pone.0124673.s004.docx]

Table S1. The compositions of 7 crystal structures of CDK9/cyclin T1 complex.

| PDB ID | Composition | Salt bridges between Arg65 and pThr186 |
| --- | --- | --- |
| 3MIA | pCDK9/cyclin T1, HIV-1 Tat, AMP-PNP | 2 |
| 3MI9 | pCDK9/cyclin T1, HIV-1 Tat, | 2 |
| 4OGR | pCDK9/cyclin T1, HIV-1 Tat, AFF4, adenosine | 2 |
| 4OR5 | pCDK9/cyclin T1, HIV-1 Tat, AFF4, | 2 |
| 4IMY | pCDK9/cyclin T1, AFF4, AMP | 1 |
| 3TNH | pCDK9/cyclin T1, CAN508 | 1 |
| 3TNI | pCDK9/cyclin T1 | 0 |
