# Supplementary material for: Determining the Functions of HIV-1 Tat and a Second Magnesium Ion in the CDK9/Cyclin T1 Complex: A Molecular Dynamics Simulation Study
Source: PLoS One. 2015 Apr 24;10(4):e0124673. doi: 10.1371/journal.pone.0124673 (PMC4409394; doi:10.1371/journal.pone.0124673)
Supplement: S2 Table — (DOCX) [file pone.0124673.s005.docx]

Table S2. Kinase crystal structures containing 2 metal ions and ATP analog.

| PDB ID | Protein | ATP analog | Metal ions | Release date |
| --- | --- | --- | --- | --- |
| 1ATP | Catalytic subunit of cAMP-dependent kinase | ATP | 2Mn^2+^ | 1993.04.15 |
| 1L3R | Catalytic subunit of cAMP-dependent kinase | ADP/AlF_3_ | 2Mg^2+^ | 2002.03.20 |
| 4IAC | Catalytic subunit of cAMP-dependent kinase | AMP-PCP | 2Mg^2+^ | 2013.06.12 |
| 4IAD | Catalytic subunit of cAMP-dependent kinase | ADP | 2Mg^2+^ | 2013.06.12 |
| 4IAF | Catalytic subunit of cAMP-dependent kinase | ADP | 2Mg^2+^ | 2013.06.12 |
| 3QHR | cyclin-dependent kinase 2 | ADP/MgF_3_^-^ | 2Mg^2+^ | 2011.05.25 |
| 3QHW | cyclin-dependent kinase 2 | ADP/MgF_3_^-^ | 2Mg^2+^ | 2011.05.25 |
| 4I3Z | cyclin-dependent kinase 2 | ADP | 2Mg^2+^ | 2012.12.26 |
| 1PYX | GSK3β | AMP-PNP | 2Mg^2+^ | 2003.10.23 |
| 1O6L | Protein kinase B | AMP-PNP | 2Mn^2+^ | 2002.11.19 |
| 1CM8 | p38γ | AMP-PNP | 2Mg^2+^ | 2000.05.17 |
| 3A7J | MST3 kinase | ADP | 2Mn^2+^ | 2012.02.02 |
